# A Multi-Parametric Radiomics Nomogram for Preoperative Prediction of Microvascular Invasion Status in Intrahepatic Cholangiocarcinoma

**DOI:** 10.3389/fonc.2022.838701

**Published:** 2022-02-24

**Authors:** Xianling Qian, Xin Lu, Xijuan Ma, Ying Zhang, Changwu Zhou, Fang Wang, Yibing Shi, Mengsu Zeng

**Affiliations:** ^1^ Department of Radiology, Zhongshan Hospital, Fudan University, Shanghai, China; ^2^ Shanghai Institute of Medical Imaging, Shanghai, China; ^3^ Department of Cancer Center, Zhongshan Hospital, Fudan University, Shanghai, China; ^4^ Department of Radiology, Xuzhou Central Hospital, Xuzhou Clinical School of Xuzhou Medical University, Xuzhou, China; ^5^ Department of Research and Development, Shanghai United Imaging Intelligence Co., Ltd., Shanghai, China

**Keywords:** intrahepatic cholangiocarcinoma, microvascular invasion, prognosis, magnetic resonance imaging, radiomics, nomogram

## Abstract

**Background:**

Intrahepatic cholangiocarcinoma (ICC) is the second most common primary liver cancer with increasing incidence in the last decades. Microvascular invasion (MVI) is a poor prognostic factor for patients with ICC, which correlates early recurrence and poor prognosis, and it can affect the selection of personalized therapeutic regime.

**Purpose:**

This study aimed to develop and validate a radiomics-based nomogram for predicting MVI in ICC patients preoperatively.

**Methods:**

A total of 163 pathologically confirmed ICC patients (training cohort: *n* = 130; validation cohort: *n* = 33) with postoperative Ga-DTPA-enhanced MR examination were enrolled, and a time-independent test cohort (*n* = 24) was collected for external validation. Univariate and multivariate analyses were used to determine the independent predictors of MVI status, which were then incorporated into the MVI prediction nomogram. Least absolute shrinkage and selection operator logistic regression was performed to select optimal features and construct radiomics models. The prediction performances of models were assessed by receiver operating characteristic (ROC) curve analysis. The performance of the MVI prediction nomogram was evaluated by its calibration, discrimination, and clinical utility.

**Results:**

Larger tumor size (*p* = 0.003) and intrahepatic duct dilatation (*p* = 0.002) are independent predictors of MVI. The final radiomics model shows desirable and stable prediction performance in the training cohort (AUC = 0.950), validation cohort (AUC = 0.883), and test cohort (AUC = 0.812). The MVI prediction nomogram incorporates tumor size, intrahepatic duct dilatation, and the final radiomics model and achieves excellent predictive efficacy in training cohort (AUC = 0.953), validation cohort (AUC = 0.861), and test cohort (AUC = 0.819), fitting well in calibration curves (*p* > 0.05). Decision curve and clinical impact curve further confirm the clinical usefulness of the nomogram.

**Conclusion:**

The nomogram incorporating tumor size, intrahepatic duct dilatation, and the final radiomics model is a potential biomarker for preoperative prediction of the MVI status in ICC patients.

## 1 Introduction

Intrahepatic cholangiocarcinoma (ICC) is the second most common primary liver cancer and accounts for 10%–15% of all cases, which arises from cholangiocytes of intrahepatic bile ducts or bile ductules (1–3). ICC has three predominant macroscopic growth patterns: mass-forming type, periductal infiltrating type, and intraductal papillary type (4). Several studies have reported increasing incidence of ICC in the last decades (5, 6) and the 5-year survival rate is still lower than 10% (7). At present, hepatectomy is still the most effective treatment for long-term survival of ICC patients (8, 9), and several poor prognostic factors have been reported, including lymph node metastasis, microvascular invasion (MVI), tumor size ≥5 cm, and multiple nodules (10).

MVI is an important histopathological feature and refers to the cancer cell nest in vessels of the surrounding hepatic tissue lined with endothelial cells (11). As a poor prognostic factor, MVI correlates early recurrence and poor outcomes and is an independent factor for overall survival in ICC patients (12, 13). However, the status of MVI is difficult to detect by radiographic images and can only be determined by histological evaluation after hepatectomy (14). In addition, MVI can affect the selection of personalized therapeutic regime, for instance, ICC patients without MVI do not need to receive adjuvant chemotherapy after R0 resections (10). Herein, the preoperative determination of MVI status is of great value in ICC patients, and it holds promises for effective patient management and estimation of outcomes.

Radiomics is a powerful and sophisticated image mining tool, and it can improve diagnostic accuracy and predict prognosis by high-throughput selecting imaging features from medical images (15). Also, several studies have constructed radiomics-based nomogram in distinguishing different pathological types of primary liver cancer (16) and predicting MVI of hepatocellular carcinoma preoperatively (17, 18). Recently, radiomics nomograms have been established for the prediction of lymph node metastasis (19), early recurrence (20), and prognosis after hepatectomy (21) in ICC patients. For MVI prediction of ICC, Zhou et al. showed the promise of seven wavelet features extracted from preoperative dynamic contrast-enhanced (DCE) MR images with an area under curve (AUC) of 0.873 (22).

In the present study, we focused on mass-forming ICCs and aimed to develop and validate a radiomics nomogram integrating clinical, imaging, and radiomics features for preoperative prediction of MVI in ICC. In order to verify the accuracy, the radiomics nomogram will be further validated by a test cohort.

## 2 Materials and Methods

### 2.1 Patients

**Figure 1:**
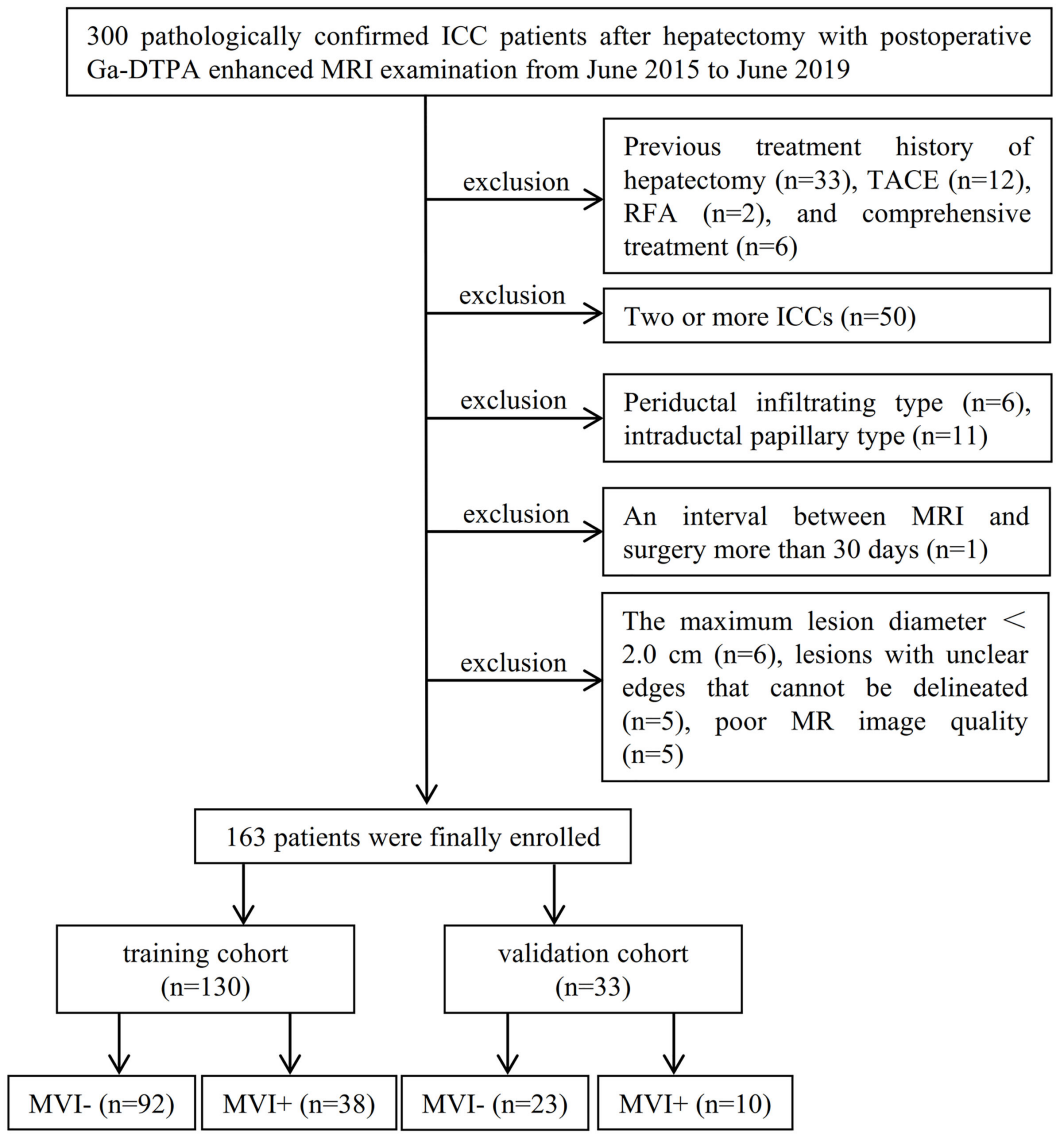
Study flowchart of the enrolled patients.

Zhongshan Hospital, Fudan University, and Xuzhou Central Hospital ethics committees approved this retrospective study, and patient informed consent was waived. Between June 2015 and June 2019, 163 pathologically confirmed ICC patients (118 men and 45 women; 60.48 ± 11.42 years) after hepatectomy with postoperative Ga-DTPA-enhanced MRI examination from Zhongshan Hospital were enrolled by the following inclusion criteria (**Figure 1**): (a) without previous history of liver cancer treatment (including hepatectomy, transcatheter arterial chemoembolization, radiofrequency ablation, chemotherapy, radiotherapy, and immunosuppressive therapy); (b) single mass-forming type ICC with the longest diameter ≥1.0 cm, and without macrovascular invasion and lymphatic metastasis; (c) complete histopathologic description of ICC; (d) MRI scanned within 30 days before surgery; (e) sufficient MR image quality satisfied the diagnostic criteria. All enrolled patients from June 2015 to June 2019 were divided into training cohort (*n* = 130, 38 MVI positive and 92 MVI negative) and validation cohort (*n* = 33, 10 MVI positive and 23 MVI negative) in a ratio of 8:2. Importantly, between July 2019 and October 2021, a time-independent test cohort (*n* = 24, 12 MVI positive and 12 MVI negative) from Zhongshan Hospital and Xuzhou Central Hospital was collected for external validation (**Supplementary Table S1**).

### 2.2 Laboratory Tests and Histopathology

Demographic and preoperative laboratory indexes (**Table 1**) including serum alpha-fetoprotein (AFP), carcinoembryonic antigen (CEA), carbohydrate antigen 19-9 (CA199), hepatitis B virus (HBV), and HBV-DNA loads were collected from our electronic medical records system. ICC specimens were sampled using 7-point baseline sampling protocol (11). Pathological characteristics including tumor number, Edmondson-Steiner grade, and MVI status were assessed in consensus by two experienced abdominal pathologists. MVI was defined as the presence of tumor cell nest (the number of suspended tumor cell more than 50) in the portal vein, hepatic vein, or large capsular vessel of the surrounding hepatic tissue that was only visible by microscopy (11, 23).

**Table 1:** Comparison of MVI status and clinicoradiologic characteristics in ICC patients of training and validation cohorts.

Characteristics	Training cohort (*n* = 130)			Validation cohort (*n* = 33)			*p*-Inter
	MVI (−), (*n* = 92)	MVI (+), (*n* = 38)	*p*-Intra	MVI (−), (*n* = 23)	MVI (+), (*n* = 10)	*p*-Intra	
Clinical features							
Age (years)^a^	60.05 (11.72)	61.21 (10.32)	0.598	60.91 (11.92)	60.70 (12.91)	0.964	0.838
Gender			0.920			0.444	0.698
Female	25 (27.2)	10 (26.3)		6 (26.1)	4 (40.0)		
Male	67 (72.8)	28 (73.7)		17 (73.9)	6 (60.0)		
HBV			0.541			0.707	0.535
Negative	49 (53.3)	18 (47.4)		14 (60.9)	5 (50.0)		
Positive	43 (46.7)	20 (52.6)		9 (39.1)	5 (50.0)		
AFP			0.808			1.000	0.930
<20 ng/ml	79 (85.9)	32 (84.2)		20 (87.0)	9 (90.0)		
≥20 ng/ml	13 (14.1)	6 (15.8)		3 (13.0)	1 (10.0)		
CEA			**0.031**			1.000	0.641
<5 ng/ml	80 (87.0)	27 (71.1)		18 (78.3)	8 (80.0)		
≥ 5ng/ml	12 (13.0)	11 (28.9)		5 (21.7)	2 (20.0)		
CA199			**0.028**			0.707	0.946
<34 U/ml	58 (63.0)	16 (42.1)		14 (60.9)	5 (50.0)		
≥34 U/ml	34 (37.0)	22 (57.9)		9 (39.1)	5 (50.0)		
Edmondson-Steiner grade			**0.017**			0.109	0.777
I–II	34 (37.0)	6 (15.8)		10 (43.5)	1 (10.0)		
III–IV	58 (63.0)	32 (84.2)		13 (56.5)	9 (90.0)		
MR imaging features							
Tumor size (mm)^a^	40.92 (21.66)	59.93 (26.55)	**<0.001**	42.70 (21.79)	46.34 (19.53)	0.653	0.568
Tumor morphology			0.168			0.279	0.713
(Hemi-)spherical and oval	40 (43.5)	10 (26.3)		12 (52.2)	3 (30.0)		
Lobulated	36 (39.1)	18 (47.4)		7 (30.4)	6 (60.0)		
Irregular	16 (17.4)	10 (26.3)		4 (17.4)	1 (10.0)		
SI on T1WI			0.236			1.000	0.693
Low	91 (98.9)	36 (94.7)		22 (95.7)	10 (100.0)		
Moderate	1 (1.1)	1 (2.6)		1 (4.3)	0 (0.0)		
High	0 (0.0)	1 (2.6)		0 (0.0)	0 (0.0)		
SI on T2WI-FS			0.699			1.000	0.474
Low	1 (1.1)	0 (0.0)		0 (0.0)	0 (0.0)		
Moderate	2 (2.2)	1 (2.6)		2 (8.7)	0 (0.0)		
High	89 (96.7)	37 (97.4)		21 (91.3)	10 (100.0)		
Target sign on T2WI-FS			0.560			0.444	0.583
Negative	58 (63.0)	26 (68.4)		17 (73.9)	6 (60.0)		
Positive	34 (37.0)	12 (31.6)		6 (26.1)	4 (40.0)		
Target sign on DWI			0.552			0.707	0.701
Negative	48 (52.2)	22 (57.9)		14 (60.9)	5 (50.0)		
Positive	44 (47.8)	16 (42.1)		9 (39.1)	5 (50.0)		
Rim enhancement on AP			0.735			0.673	0.522
Negative	17 (18.5)	8 (21.1)		5 (21.7)	3 (30.0)		
Positive	75 (81.5)	30 (78.9)		18 (78.3)	7 (70.0)		
Complete rim on AP			0.288			0.378	0.580
Negative	29 (38.7)	15 (50.0)		10 (55.6)	2 (28.6)		
Positive	46 (61.3)	15 (50.0)		8 (44.4)	5 (71.4)		
Enhancement pattern			0.423			0.195	0.376
Gradual and filling	70 (76.1)	29 (76.3)		14 (60.9)	8 (80.0)		
Arterial and persistent	13 (14.1)	3 (7.9)		4 (17.4)	0 (0.00)		
Wash-in and wash-out	9 (9.8)	6 (15.8)		5 (21.7)	2 (20.0)		
LI-RADS			0.087			1.000	0.242
LR-3	1 (1.1)	0 (0.0)		0 (0.0)	0 (0.0)		
LR-4	5 (5.4)	0 (0.0)		0 (0.0)	0 (0.0)		
LR-5	4 (4.3)	4 (10.5)		4 (17.4)	1 (10.0)		
LR-M	82 (89.1)	33 (86.8)		19 (82.6)	9 (90.0)		
LR-TIV	0 (0.0)	1 (2.6)		0 (0.0)	0 (0.0)		
Intrahepatic duct dilatation			**<0.001**			0.686	0.114
Negative	64 (69.6)	11 (28.9)		16 (69.6)	8 (80.0)		
Positive	28 (30.4)	27 (71.1)		7 (30.4)	2 (20.0)		
Hepatic capsular retraction			0.806			0.139	0.702
Negative	53 (57.6)	21 (55.3)		16 (69.6)	4 (40.0)		
Positive	39 (42.4)	17 (44.7)		7 (30.4)	6 (60.0)		
Visible vessel penetration			0.599			1.000	0.618
Negative	36 (39.1)	13 (34.2)		10 (43.5)	4 (40.0)		
Positive	56 (60.9)	25 (65.8)		13 (56.5)	6 (60.0)		
Peripherally hepatic enhancement			0.146			1.000	0.351
Negative	38 (41.3)	21 (55.3)		8 (34.8)	4 (40.0)		
Positive	54 (58.7)	17 (44.7)		15 (65.2)	6 (60.0)		

Data are shown as number of patients and percentage in parentheses, unless otherwise stated.

a Data are means and standard deviations in parentheses.

MVI, microvascular invasion; OR, odds ratio; HBV, hepatitis B; AFP, α-fetoprotein; CEA, carcinoembryonic antigen; CA199, carbohydrate antigen 199; SI, signal intensity; T1WI, T1-weighted imaging; T2WI, T2-weighted imaging; FS, fat suppression; DWI, diffusion-weighted imaging; LI-RADS, the liver imaging reporting and data system; AP, arterial phase. The bold values are statistically significant with p <0.05.

### 2.3 Gd-DTPA MR Imaging

All patients underwent MR imaging with intravenous injection of 0.2 mmol/kg gadopentetate dimeglumine (Gd-DTPA; Bayer HealthCare, Berlin, Germany) and immediately followed by a 20-ml saline flush at a speed of 2 ml/s. Taking Magnetom Aera 1.5T scanner (Siemens Healthcare, Erlangen, Germany) as an example, imaging sequences included axial T2-weighted imaging with fat suppression (T2WI-FS), diffusion-weighted imaging (DWI), in-phase and opposed-phase T1-weighted imaging (IP-OP T1WI), axial precontrast three-dimensional volumetric-interpolated breath-hold examination T1-weighted imaging (3D-VIBE T1WI) with fat suppression, and postcontrast dynamic-enhanced 3D-VIBE-T1WI at arterial phase (AP, 20–30 s), portal venous phase (PVP, 60–70 s), and delayed phase (DP, 180 s). Detailed parameters of each acquisition sequence are shown in **Supplementary Table S2**.

### 2.4 MR Images Analysis

All MR images were reviewed independently on a picture archiving and communication system (PACS; Pathspeed, GE Medical Systems Integrated Imaging Solutions, Chicago, IL, USA) by 2 experienced abdominal radiologists (XM and XL with 10 and 15 years of experience, respectively). Both radiologists were blinded to all demographic, clinical, laboratory, and histopathologic information. If any discrepancies occurred, a consensus was reached after discussion. The following imaging features were assessed by 2 abdominal radiologists: (a) tumor size, defined as the maximum diameter on transverse T1WI image; (b) tumor morphology, including spherical/hemispherical/oval, lobulated and irregular; (c) signal intensity on T1WI, T2WI-FS, and DWI, including hypointense, isointense, and hyperintense; (d) target sign on T2WI-FS and DWI, defined as peripheral hyperintense with central isointense/hypointense (24); (e) rim enhancement on AP, defined as peripheral enhancement of the lesion on AP, including complete and incomplete rim; (f) enhancement pattern, including gradual and filling, arterial and persistent, and wash-in and wash-out enhancement; (g) the liver imaging reporting and data system (LI-RADS), defined as LR category based on LI-RADSv2018 (25); (h) intrahepatic duct dilatation, defined as intrahepatic duct dilatation within or outside of the lesion; (i) hepatic capsular retraction, defined as retraction of hepatic capsular adjacent to the lesion; (j) visible vessel penetration, defined as the presence of penetrating vessels in the lesion, including hepatic artery, portal vein, and hepatic vein (26); and (k) peripherally hepatic enhancement, defined as enhancement of liver parenchyma around the lesion on any phase.

### 2.5 Radiomics Analysis

#### 2.5.1 Workflow

**Figure 2:**
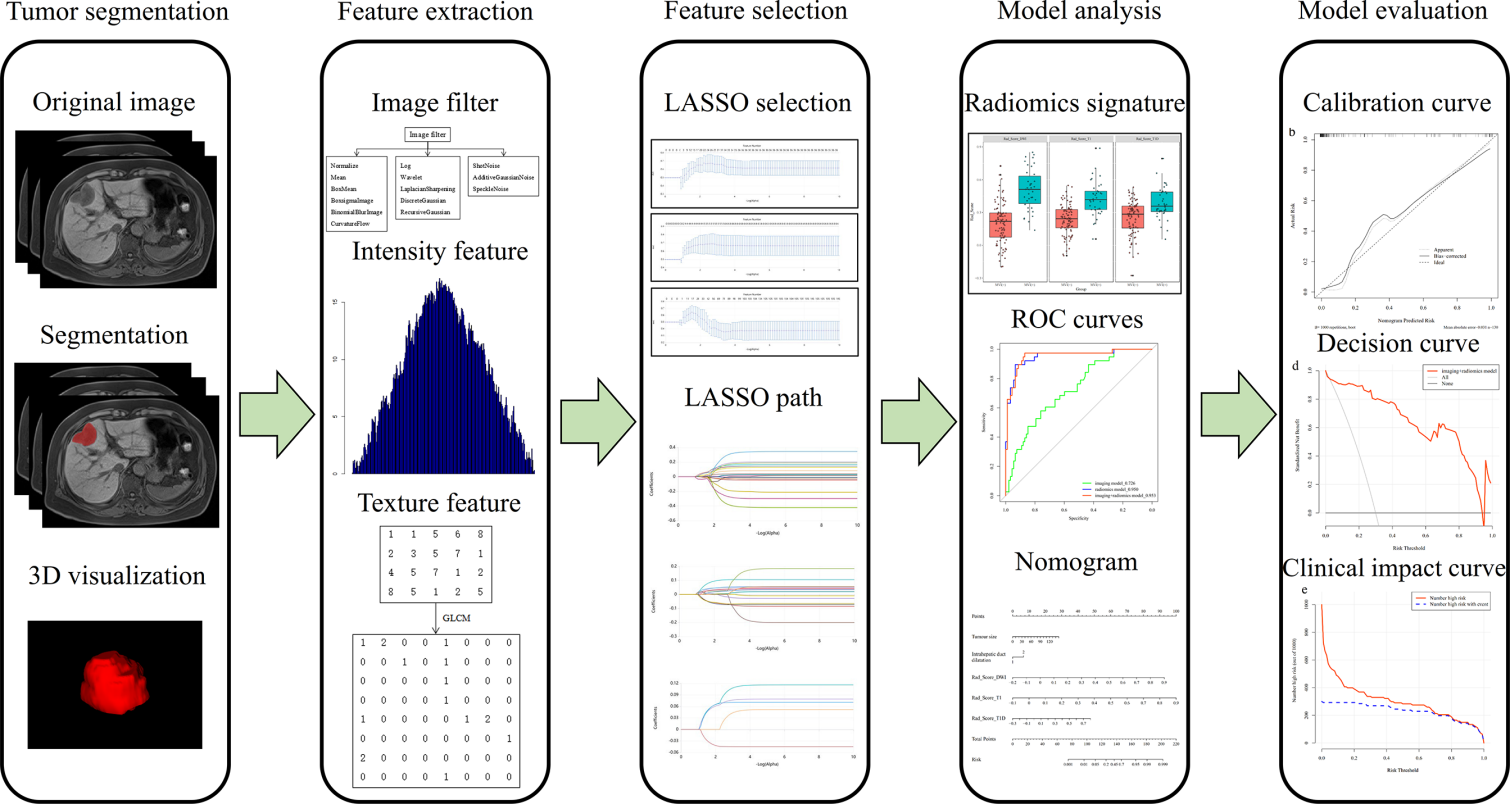
Study flowchart of the radiomics analysis.

The workflow of the radiomics analysis included tumor segmentation, feature extraction, feature selection, model construction, model analysis, and evaluation (**Figure 2**).

#### 2.5.2 Image Segmentation

The whole tumor segmentation was manually delineated in ITK-SNAP (http://www.itksnap.org/pmwiki/pmwiki.php) by an abdominal radiologist with 6 years of experience (XQ) and validated by a senior abdominal radiologist with 15 years of experience (XL). Volumes of interests (VOIs) were drawn on 6 sequences: DWI with *b*-values of 500 s/mm^2^, T2WI-FS, 3D-VIBE T1WI, AP, PVP, and DP.

#### 2.5.3 Feature Extraction

Radiomics features were extracted from the VOIs using uAI Research Portal (Version: 20210730), and 2,600 radiomics features were extracted from each sequence (**Supplementary Table S3**). These radiomics features were classified into First Order, Shape, Gray-Level Co-occurrence Matrix (GLCM), Gray-Level Size Zone Matrix (GLSZM), Gray-Level Run Length Matrix (GLRLM), Neighboring Gray Tone Difference Matrix (NGTDM), and Gray-Level Dependence Matrix (GLDM) features.

#### 2.5.4 Feature Selection

To eliminate index dimension difference, the extracted radiomics features of each sequence were standardized into a normal distribution with z-scores. A test-retest procedure was performed on 30 randomly selected tumors, reproducible radiomics features were considered features with intraclass correlation coefficient greater than 0.75 and included in the following feature selection procedures. The variance threshold, SelectKBest and least absolute shrinkage and selection operator (LASSO) methods were performed to select optimal features of each sequence (**Supplementary Table S3** and **Supplementary Figure S1**). The corresponding radiomics score (Rad-score) of each sequence in the training and validation cohorts was calculated (**Supplementary Table S4** and **Supplementary Figure S2**).

#### 2.5.5 Model Construction

To construct clinical and imaging models, the univariate analysis was used to assess the potential predictors of MVI status, and the multivariate logistic regression analysis was used to determine the independent predictors of MVI status. Radiomics models of each sequence were constructed by the corresponding optimal features. The sequences with Rad-scores showed significant differences between MVI-positive and MVI-negative ICCs in both the training cohort and validation cohort were selected for the final radiomics model construction. The MVI prediction model incorporated imaging model and the final radiomics model. All models were constructed with logistic regression (LR), random forest (RF), and support vector machine (SVM) classifiers respectively for comparison.

#### 2.5.6 Model Analysis and Evaluation

The receiver operating characteristic curves were plotted, and the AUC, accuracy, sensitivity, specificity, F1-score, and precision were calculated to quantify the predictive efficacy of each model in training, validation, and test cohorts. The comparison of predictive performances between multiple models was performed by the Delong test. A radiomics nomogram was built on the MVI prediction model with the LR classifier. Hosmer-Lemeshow test was performed to identify the agreement between nomogram-predicted MVI status and actual MVI status, and calibration curves in the training and validation cohorts were plotted. Decision curve and clinical impact curve were plotted for assessing the clinical usefulness of the nomogram by quantifying the net benefits at different risk thresholds.

### 2.6 Statistical Analysis

Clinical and imaging features were analyzed for statistical differences in the training, validation, and test cohorts by Student’s *t*-test, Mann-Whitney *U* test, Wilcoxon test, Chi-square test, or Fisher’s exact test, as appropriate. The statistical analyses were conducted using the IBM SPSS Statistics (version 20) and R software (version 3.4.1). A two-tailed *p*-value of less than 0.05 was considered statistically significant.

## 3 Results

### 3.1 Clinicoradiologic Characteristics and Performances

**Figure 3:**
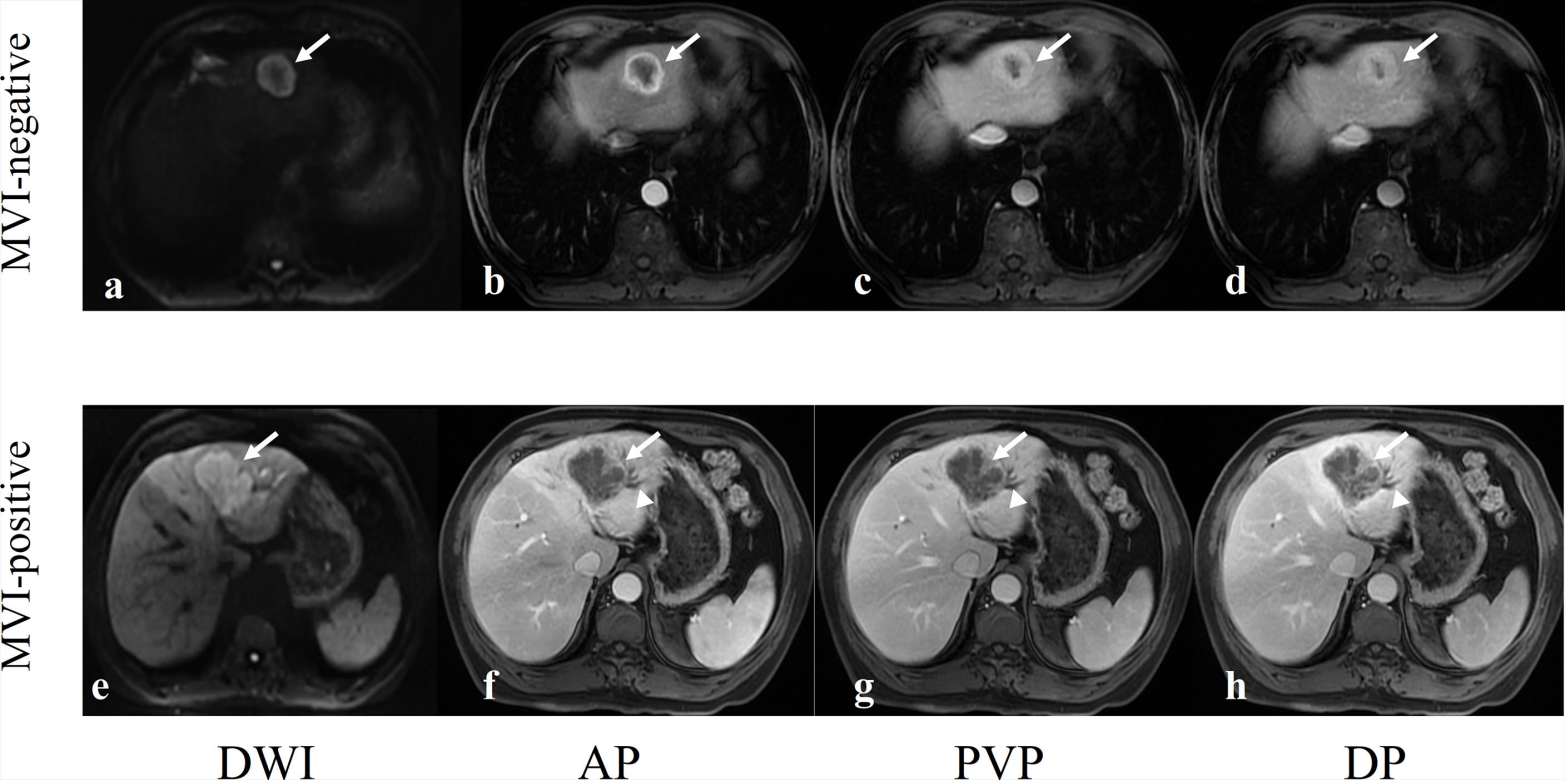
Two examples of representative MVI-negative and MVI-positive ICCs. **(A–D)** A 62-year-old man with a well-circumscribed MVI-negative ICC in hepatic segment II (arrows). DWI image showed target sign **(A)**, axial arterial phase image showed rim enhancement **(B)**, and portal vein phase image **(C)** and delayed phase image **(D)** showed the typical enhancement type of ICC: gradual and filling enhancement. **(E–H)** A 62-year-old man with a lobulated MVI-positive ICC in hepatic segment IV (arrows). DWI image showed hyperintensity **(E)**, axial arterial phase image showed marginal moderate enhancement with no internal enhancement **(F)** and dilated bile ducts next to tumor (arrowheads), and portal vein phase image **(G)** and delayed phase image **(H)** showed the typical enhancement type of ICC: gradual and filling enhancement.

Comparison of MVI status and clinicoradiologic characteristics in training and validated ICC patients are shown in **Table 1**. The final cohort of 163 patients with single ICC was divided into training cohort (*n* = 130, 92 patients were MVI negative and 38 patients were MVI positive) and validation cohort (*n* = 33, 23 patients were MVI negative and 10 patients were MVI positive). There is no significant difference of the status of MVI between training and validation cohort (*p* = 0.904). Univariate analysis of clinicoradiologic characteristics indicates that serum CEA level (*p* = 0.035; OR = 2.716, 95% CI: 1.065–6.918), serum CA199 level (*p* = 0.030; OR = 2.346, 95% CI: 1.092–5.139), Edmondson-Steiner grade (*p* = 0.021; OR = 3.126, 95% CI: 1.254–8.977), tumor size (*p* < 0.001; OR = 1.033, 95% CI: 1.016–1.052), tumor morphology (*p*= 0.071; OR = 1.604, 95% CI: 0.964–2.708), and intrahepatic duct dilatation (*p* < 0.001; OR =5.610, 95% CI: 2.505–13.308) are significantly associated with MVI. At the multivariate analysis, tumor size (*p* = 0.003; OR = 1.032, 95% CI: 1.011–1.055) and intrahepatic duct dilatation (*p* = 0.002; OR = 4.552, 95% CI: 1.777–12.259) are independent predictors of MVI (**Table 2**). The imaging model constructed with two predictors has an AUC of 0.726 in the training cohort and 0.522 in the validation cohort (**Table 3**). Examples of representative radiological characteristics of MVI-positive and MVI-negative ICCs are shown in **Figure 3**.

**Table 2:** Univariate and multivariate analyses of predictive characteristics related with MVI status in ICC.

Characteristics	Univariate		Multivariate	
	*p*-value	OR (95% CI)	*p*-value	OR (95% CI)
Age	0.595	1.009 (0.976–1.045)		
Gender	0.920	0.957 (0.394–2.211)		
HBV	0.541	1.266 (0.594–2.717)		
AFP	0.808	1.139 (0.373–3.157)		
CEA	**0.035**	2.716 (1.065–6.918)	0.463	1.517 (0.491–4.629)
CA199	**0.030**	2.346 (1.092–5.139)	0.973	0.984 (0.365–2.541)
Edmondson-Steiner grade	**0.021**	3.126 (1.254–8.977)		
Tumor size	**<0.001**	1.033 (1.016–1.052)	**0.003**	1.032 (1.011–1.055)
Tumor morphology	**0.071**	1.604 (0.964–2.708)	0.440	0.757 (0.362–1.504)
SI on T1WI	0.175	4.225 (0.715–80.403)		
SI on T2WI-FS	0.701	1.455 (0.282–24.396)		
Target sign on T2WI-FS	0.541	0.776 (0.337–1.723)		
Target sign on DWI	0.552	0.793 (0.366–1.695)		
Rim enhancement on AP	0.735	0.850 (0.339–2.273)		
Enhancement pattern	0.659	1.130 (0.640–1.926)		
LI-RADS	0.715	0.912 (0.521–1.447)		
Intrahepatic duct dilatation	**<0.001**	5.610 (2.505–13.308)	**0.002**	4.552 (1.777–12.259)
Hepatic capsular retraction	0.806	1.100 (0.510–2.355)		
Visible vessel penetration	0.599	1.236 (0.567–2.780)		
Peripherally hepatic enhancement	0.148	0.570 (0.263–1.217)		

The bold values are statistically significant with p <0.05.

**Table 3:** The performance of imaging, radiomics of single and multiple MR sequences, and final fusion models for predicting MVI in ICC patients.

Models	Classifier and cohort	AUC	Accuracy	Sensitivity	Specificity	Precision
Imaging model	LR (TD/VD)	0.726/0.522	0.669/0.545	0.605/0.400	0.696/0.609	0.451/0.308
	RF (TD/VD)	0.742/0.578	0.731/0.697	0.211/0.100	0.946/0.957	0.615/0.500
	SVM (TD/VD)	0.726/0.483	0.708/0.697	0.000/0.000	1.000/1.000	0.000/0.000
DWI model	LR (TD/VD)	1.000/0.530	1.000/0.485	1.000/0.600	1.000/0.435	1.000/0.316
	RF (TD/VD)	0.943/0.530	0.800/0.697	0.316/0.000	1.000/1.000	1.000/0.000
	SVM (TD/VD)	1.000/0.774	1.000/0.697	1.000/0.000	1.000/1.000	1.000/0.000
T1 model	LR (TD/VD)	1.000/0.643	1.000/0.636	1.000/0.700	1.000/0.609	1.000/0.438
	RF (TD/VD)	0.949/0.687	0.823/0.697	0.395/0.100	1.000/0.957	1.000/0.500
	SVM (TD/VD)	1.000/0.513	1.000/0.697	1.000/0.000	1.000/1.000	1.000/0.000
T1A model	LR (TD/VD)	1.000/0.443	1.000/0.636	1.000/0.500	1.000/0.304	1.000/0.238
	RF (TD/VD)	0.967/0.700	1.000/0.364	0.158/0.000	1.000/1.000	1.000/0.000
	SVM (TD/VD)	1.000/0.500	0.754/0.697	1.000/0.000	1.000/1.000	1.000/0.000
T1D model	LR (TD/VD)	1.000/0.665	1.000/0.606	1.000/0.700	1.000/0.565	1.000/0.412
	RF (TD/VD)	0.978/0.765	0.738/0.697	0.105/0.000	1.000/1.000	1.000/0.000
	SVM (TD/VD)	1.000/0.574	1.000/0.697	1.000/0.000	1.000/1.000	1.000/0.000
T1V model	LR (TD/VD)	1.000/0.430	1.000/0.424	1.000/0.600	1.000/0.348	1.000/0.286
	RF (TD/VD)	0.979/0.661	0.738/0.697	0.105/0.000	1.000/1.000	1.000/0.000
	SVM (TD/VD)	1.000/0.426	1.000/0.697	1.000/0.000	1.000/1.000	1.000/0.000
T2 model	LR (TD/VD)	1.000/0.422	1.000/0.424	1.000/0.100	1.000/0.565	1.000/0.091
	RF (TD/VD)	0.969/0.383	0.746/0.697	0.132/0.000	1.000/1.000	1.000/0.000
	SVM (TD/VD)	1.000/0.448	1.000/0.697	1.000/0.000	1.000/1.000	1.000/0.000
DWI+T1 model	LR (TD/VD)	0.941/0.817	0.892/0.758	0.895/0.800	0.891/0.739	0.773/0.571
	RF (TD/VD)	0.963/0.854	0.908/0.848	0.895/0.900	0.913/0.826	0.810/0.692
	SVM (TD/VD)	0.941/0.826	0.892/0.788	0.816/0.800	0.924/0.783	0.816/0.615
DWI+T1D model	LR (TD/VD)	0.901/0.852	0.846/0.788	0.684/0.700	0.913/0.826	0.765/0.636
	RF (TD/VD)	0.897/0.852	0.792/0.636	0.816/0.800	0.783/0.565	0.608/0.444
	SVM (TD/VD)	0.890/0.835	0.815/0.788	0.474/0.600	0.957/0.870	0.818/0.667
T1+T1D model	LR (TD/VD)	0.883/0.874	0.846/0.818	0.711/0.600	0.902/0.913	0.705/0.750
	RF (TD/VD)	0.905/0.878	0.869/0.818	0.816/0.800	0.891/0.826	0.756/0.667
	SVM (TD/VD)	0.884/0.835	0.777/0.727	0.237/0.100	1.000/1.000	1.000/1.000
Radiomics model	LR (TD/VD)	0.950/0.883	0.862/0.788	0.921/0.900	0.837/0.739	0.700/0.600
	RF (TD/VD)	0.967/0.891	0.908/0.879	0.895/0.900	0.913/0.870	0.801/0.750
	SVM (TD/VD)	0.947/0.865	0.869/0.818	0.579/0.700	0.989/0.870	0.957/0.700
Imaging+radiomics model	LR (TD/VD)	0.953/0.861	0.892/0.818	0.974/0.900	0.859/0.783	0.740/0.643
	RF (TD/VD)	0.988/0.878	0.946/0.909	0.895/0.800	0.967/0.957	0.919/0.889
	SVM (TD/VD)	0.898/0.878	0.869/0.909	0.763/0.900	0.913/0.913	0.784/0.818
Radiomics model	LR (test)	0.812 (0.617–1.000)	0.792	0.750	0.833	0.818
	RF (test)	0.757 (0.532–0.982)	0.792	0.667	0.917	0.889
	SVM (test)	0.812 (0.617–1.000)	0.708	0.500	0.917	0.857
Imaging+radiomics model	LR (test)	0.819 (0.620–1.000)	0.875	0.833	0.917	0.909
	RF (test)	0.771 (0.556–0.986)	0.750	0.583	0.917	0.875
	SVM (test)	0.771 (0.555–0.987)	0.792	0.667	0.917	0.889

LR, logistic regression; RF, random forest; SVM, support vector machine; TD, training dataset; VD, validation dataset; AUC, area under the curve.

### 3.2 Performance of Radiomics Features From Single MR Sequence

Robust radiomics features of each single MR sequence were selected by reproducibility analysis and LASSO analysis (**Supplementary Table S3** and **Supplementary Figure S1**). The predictive performance of radiomics features on each MR sequence is shown in **Table 3**. However, all single sequences show overfit predictive performance in the training cohort (AUCs = 1.000) and poor predictive performance in the validation cohort (AUCs: 0.422–0.665). The Rad-score of each MR sequence in the training and validation cohorts are shown in **Supplementary Table S4**, and Rad-scores of the diffusion-weighted imaging, precontrast T1-weighted imaging, and delayed phase imaging show significant differences between MVI-positive and MVI-negative ICCs in both the training cohort (*p* < 0.001) and validation cohort (*p*_DWI_ = 0.025, *p*_T1_ = 0.003, *p*_T1D_ = 0.001) (**Supplementary Table S4** and **Supplementary Figure S2**). Therefore, further analysis about diffusion-weighted imaging, precontrast T1-weighted imaging, and delayed phase imaging was conducted. The detailed information of 22 radiomics features in diffusion-weighted imaging, 17 radiomics features in precontrast T1-weighted imaging, and 5 radiomics features in delayed phase imaging are shown in **Supplementary Table S5**.

### 3.3 Performance of Radiomics Features From Multiple MR Sequences

Three pairwise combination models of the diffusion-weighted images, precontrast T1-weighted images, and delayed phase images are constructed, and all show a satisfying performance in both the training cohort (AUC = 0.883–0.941) and validation cohort (AUC = 0.817–0.874) (**Table 3**). The final radiomics model incorporates these three sequences, and it performs better in predicting MVI of ICC in both the training cohort (AUC = 0.950, accuracy = 0.862, sensitivity = 0.921, and specificity = 0.837) and validation cohort (AUC = 0.883, accuracy = 0.788, sensitivity = 0.900, and specificity = 0.739) than three pairwise combination models (**Tables 3**, **4**). Notably, the final radiomics model also performs desirably and stably in the test cohort with AUC, accuracy, sensitivity, and specificity of 0.812 (95% CI: 0.617–1.000), 0.792, 0.750, and 0.833, respectively (**Table 3**).

**Table 4:** The comparison of models in training, validation, and test cohorts.

Models for comparison	Classifier	*p*_training cohort_	*p*_validation cohort_	*p*_test cohort_
Radiomics model vs. DWI+T1 model	LR	0.222	0.013	0.591
	RF	0.674	0.217	0.260
	SVM	0.636	0.197	0.151
Radiomics model vs. DWI+T1D model	LR	0.014	0.527	0.766
	RF	0.003	0.522	0.493
	SVM	0.012	0.421	0.214
Radiomics model vs. T1+T1D model	LR	0.018	0.888	0.092
	RF	0.006	0.751	0.659
	SVM	0.027	0.572	0.334
Radiomics model vs. imaging model	LR	<0.001	0.018	0.193
	RF	<0.001	0.023	0.071
	SVM	<0.001	0.003	0.294
Imaging+radiomics model vs. imaging model	LR	<0.001	0.023	0.206
	RF	<0.001	0.021	0.055
	SVM	<0.001	0.002	0.306
Imaging+radiomics model vs. radiomics model	LR	0.629	0.202	0.732
	RF	0.032	0.505	0.569
	SVM	0.018	0.757	0.325

### 3.4 Performance of MVI Prediction Model

The MVI prediction model includes imaging model and final radiomics model, and it achieves excellent predictive efficacy in the training cohort (AUC = 0.953, accuracy = 0.892, sensitivity = 0.974, and specificity = 0.859), validation cohort (AUC = 0.861, accuracy = 0.818, sensitivity = 0.900, and specificity = 0.783), and test cohort (AUC = 0.819, accuracy = 0.875, sensitivity = 0.833, and specificity = 0.917) (**Table 3**).

**Figure 4:**
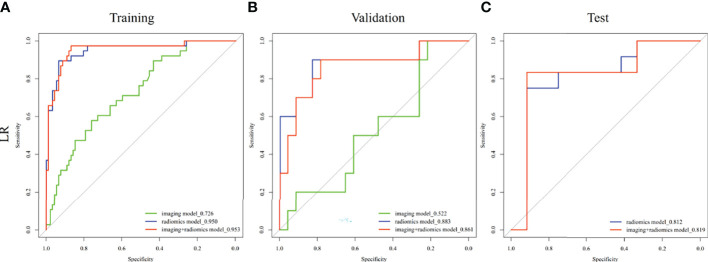
Comparison of receiver operating characteristic (ROC) curves for prediction of MVI in ICC. ROC curves of imaging model constructed with tumor size and intrahepatic duct dilatation, radiomics model constructed with diffusion-weighted image, precontrast T1-weighted image, and delayed phase image, and MVI prediction model constructed imaging model and radiomics model in the **(A)** training, **(B)** validation, and **(C)** test cohorts.

Moreover, the MVI prediction model performs better than imaging model in the training cohort (AUCs: 0.953 vs. 0.726, *p* < 0.001) and validation cohort (AUCs: 0.861 vs. 0.522, *p* = 0.018). However, there is no statistical difference between the MVI prediction model and final radiomics model in the training cohort (AUCs: 0.953 vs. 0.950, *p* = 0.629), validation cohort (AUCs: 0.861 vs. 0.883, *p* = 0.202), and test cohort (AUCs: 0.819 vs. 0.812, *p* = 0.732) (**Table 4**, **Figure 4** and **Supplementary Figure S3**).

### 3.5 Development and Verification of the Nomogram

**Figure 5:**
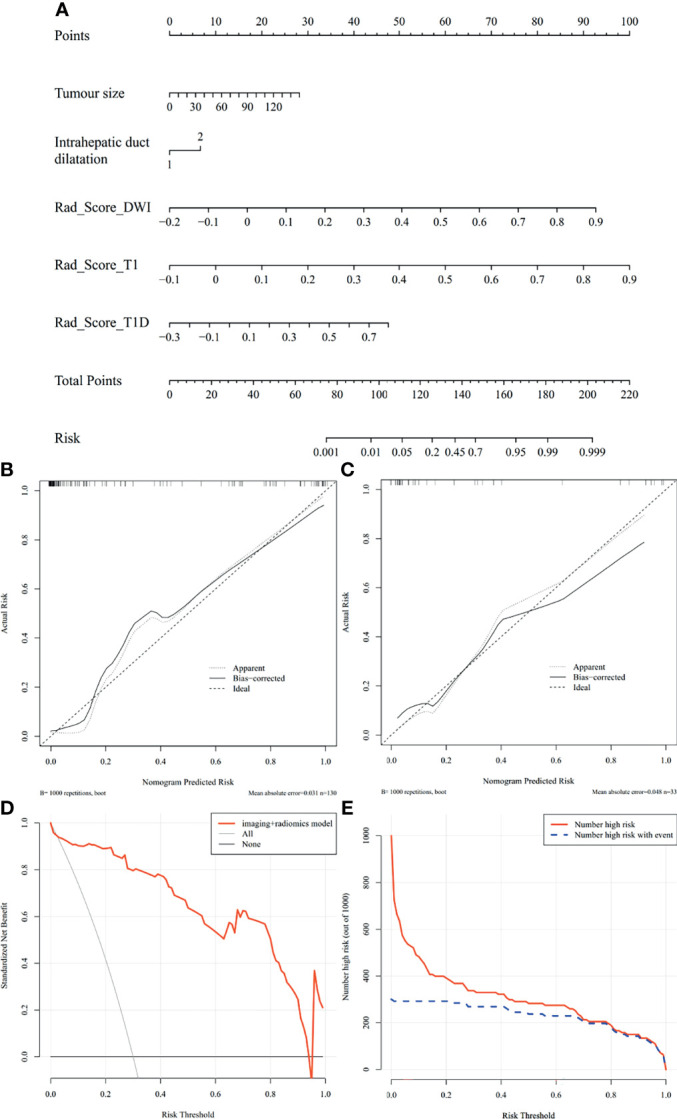
Nomogram of MVI prediction model, calibration curves of the nomogram in the training and validation cohort, decision curve, and clinical impact curve in the overall ICC patients. **(A)** A nomogram integrates imaging factors including tumor size and intrahepatic duct dilatation, and radiomics factors includes Rad-scores of diffusion-weighted images, precontrast T1-weighted images, and delayed phase images. **(B, C)** Calibration curves of the nomogram in the training and validation cohort. *x*-axis is a nomogram-predicted risk of MVI. *y*-axis is actual risk of MVI, and the diagonal dashed line indicates the ideal prediction by an ideal model. **(D)** Decision curve for the nomogram in the overall patients. The gray line is the net benefit of assuming that all patients have MVI; the black line is the net benefit of assuming no patients have MVI; and the red line is the expected net benefit of per patient based on the nomogram. **(E)** Clinical impact curve for the nomogram in the 1,000 simulated samples. The blue dashed line is the actual number of high risk, and the red line is the number of high risk based on nomogram.

The nomogram of the MVI prediction model is presented in **Figure 5A**, and the formula is as follows. It achieves satisfying performance with AUCs of 0.953 in the training cohort and 0.861 in the validation cohort. Calibration curves (**Figures 5B, C**) of the nomogram exhibiting satisfactory predictive performances are aligned with the actual MVI estimates in the training (*p* = 0.364) and validation (*p* = 0.543) cohorts. Decision curve (**Figure 5D**) of the nomogram shows that the net benefit is higher than that assuming all patients have MVI. Clinical impact curve (**Figure 5E**) shows that the predicted probabilities of the nomogram is close to actual high risk with event when risk threshold is 0.2–0.7 and is consistent when risk threshold is over 0.7.


$$Y=-11.420+0.020\times {\text{Imaging}}_{\text{Tumor size}}+0.723\times {\text{Imaging}}_{\text{Intrahepatic duct dilatation}}+9.130\times {\text{Rad Score}}_{\text{DWI}}+10.835\times {\text{Rad Score}}_{\text{T}1}+4.690\times {\text{Rad Score}}_{\text{T}1\text{D}}$$

## Discussion

In this study, we established a radiomics-based nomogram for preoperatively predicting MVI in patients with ICC. The final MVI prediction model achieves a satisfying prediction performance, and it incorporates Ga-DTPA-enhanced MRI-based radiomics features of the diffusion-weighted images, precontrast T1-weighted images, and delayed phase images and imaging features including tumor size and intrahepatic duct dilatation.

High serum level of CEA and CA199 (>100 U/ml) can predict the diagnosis of ICC (27, 28), but the predictive value in predicting MVI status of ICC is still unknown. Although univariate analysis shows elevated serum CEA and CA199 level are significant clinical features, there are no statistical difference between the MVI-positive and MVI-negative groups by multivariate analysis, which are consistent with the studies of Zhou et al. (22) and Ma et al. (29). As other studies on preoperative MVI prediction in HCC (30–32) and ICC (14, 22, 33), tumor size is also an independent predictor of MVI in ICC patients in our study, but intrahepatic duct dilatation is another important imaging predictor, which is different from the studies of Zhou et al. (22). This may be due to the different MVI predictor screening methods; in his study, intrahepatic duct dilatation was excluded because there was no significant difference between MVI-positive and MVI-negative groups in the validation cohort (*p* = 0.279), but a *p*-value with 0.097 in the training cohort indicated this predictor should be further analyzed by multivariate analysis. Although hepatic capsular retraction and progressive centripetal enhancement in the venous phase have been described as classical imaging features of ICC (34), there are no statistical difference between MVI-positive and MVI-negative groups. In general, the imaging model constructed with tumor size and intrahepatic duct dilatation yields a good performance in the training cohort, but an unsatisfying performance in the validation cohort limits its application in preoperatively predicting MVI. Therefore, a combined model based on radiomics is necessary.

Of 44 radiomics features, 6 are considered optimal features with the absolute values of LASSO coefficients being greater than 0.1, including First Order _Uniformity, GLCM _Maximum Probability, GLCM _Inverse Difference, GLCM _ Informational Measure of Correlation 1, GLDM _Dependence Variance, and GLRLM _Long Run Emphasis. Histologically, ICC often shows aggressive trait, and inflammation, necrosis, and fibrosis are common (2); therefore, heterogeneous signal intensity in MR images are found to be more frequent for MVI-positive ICC, which is in concordance with First Order _Uniformity. Additionally, other 5 radiomics features indicate the higher the neighboring intensity value, variance, and gray-level value of VOI, the higher is the probability of MVI. Compared with the 100 radiomics features pool constructed by Zhou et al. (22), 42 of 44 radiomics features in our study are repeatable, and 3 of 7 optimal radiomics features in his study are also detected in our study. Both three pairwise combination models and the final radiomics model based on the diffusion-weighted images, precontrast T1-weighted images, and delayed phase images have solved the defect of overfit predictive performance in the training cohort and poor predictive performance in the validation cohort of single MR sequence models.

As far as we know, this study is the first to establish a nomogram based on clinicoradiologic and radiomics features. The MVI prediction model incorporates the final radiomics model, and imaging model exhibits excellent performance in both the training cohort (AUC = 0.953) and validation cohort (AUC = 0.861) with good calibration, which are better than the previous study (AUC_training_ = 0.873, AUC_validation_ = 0.850) (22). Also, the MVI prediction model achieves better efficacy than the imaging model in our study, suggesting radiomics features are indispensable in MVI prediction. More importantly, the nomogram in our study also exhibits a desirable prediction performance in the test cohort (AUC = 0.819) and performs better than clinical factor model constructed by Tang et al. (35) with AUCs of 0.739, 0.717, and 0.709 in training, validation, and test cohorts. Hence, the use of our nomogram preoperatively may be a noninvasive and robust method of assisting personalized treatment making, and patients with ICCs may suffer a higher net benefit from it.

There are some limitations in our study. Firstly, selection bias is inevitable in our retrospective study. Secondly, compared with MVI-negative ICCs, the number of MVI-positive ICCs is relatively small. Thirdly, the association between radiomics features and complex tumor biological features needs to be further explained. Fourthly, the study about whether the preoperative prediction of overall survival (OS) and disease-free survival (DFS) based on our MVI prediction model has the same effect as the postoperative prediction of OS and DFS based on pathological MVI after surgery is needed. Finally, although the nomogram has achieved a desirable prediction performance in the test cohort, larger cohorts from other centers are needed to be collected for the prospective validation of our nomogram.

In conclusion, radiomics features extracted from diffusion-weighted images, precontrast T1-weighted images, and delayed phase images of Ga-DTPA-enhanced MR imaging can assist in predicting MVI status of ICC patients. The MVI prediction nomogram incorporating radiomics features and imaging features including tumor size and intrahepatic duct dilatation is a potential biomarker and clinical tool in MVI stratification of ICC patients preoperatively.

## Data Availability Statement

The original contributions presented in the study are included in the article/**Supplementary Material**. Further inquiries can be directed to the corresponding authors.

## Ethics Statement

The studies involving human participants were reviewed and approved by the Zhongshan Hospital, Fudan University ethics committee and Xuzhou Central Hospital ethics committee. The patients/participants provided their written informed consent to participate in this study. Written informed consent was obtained from the individual(s) for the publication of any potentially identifiable images or data included in this article.

## Author Contributions

Conceptualization: MZ and YS. Methodology: MZ, XQ, and FW. Software: XQ, XL, and FW. Validation: XM, YZ, and CZ. Formal analysis: XQ and XL. Resources: XQ, XL, and MZ. Data curation: XQ and XL. Writing—original draft preparation: XQ and XL. Writing—review and editing: MZ and YS. Supervision: MZ and YS. Project administration: MZ. Funding acquisition: MZ and YS. All authors listed have made a substantial, direct, and intellectual contribution to the work and approved it for publication.

## Funding

This study was supported by grants from the National Natural Science Foundation of China (No. 82171897), Shanghai Municipal Key Clinical Specialty (No. shslczdzk03202), Clinical Research Plan of SHDC (No. SHDC2020CR1029B), Clinical Research Project of Zhongshan Hospital, Fudan University (No. 2020ZSLC61), “the Development Research Foundation of Affiliated Hospital” of Xuzhou Medical University (No. XYFM2020020), and the Medical Scientific Research Program of Jiangsu Commission of Health (No. M2021014).

## Conflict of Interest

Author FW is employed by Shanghai United Imaging Intelligence Co., Ltd. Shanghai, China.

The remaining authors declare that the research was conducted in the absence of any commercial or financial relationships that could be construed as a potential conflict of interest.

## Publisher’s Note

All claims expressed in this article are solely those of the authors and do not necessarily represent those of their affiliated organizations, or those of the publisher, the editors and the reviewers. Any product that may be evaluated in this article, or claim that may be made by its manufacturer, is not guaranteed or endorsed by the publisher.
