# Supplementary material for: A Multi-Parametric Radiomics Nomogram for Preoperative Prediction of Microvascular Invasion Status in Intrahepatic Cholangiocarcinoma
Source: Front Oncol. 2022 Feb 24;12:838701. doi: 10.3389/fonc.2022.838701 (PMC8907475; doi:10.3389/fonc.2022.838701)
Supplement: Supplementary file 1 [file DataSheet_1.docx]

Supplementary Material

**Supplementary Table 1.** comparison of MVI status and clinicoradiological characteristics in ICC patients of test cohort.

| Characteristics | Test cohort (*n*=24) | | |
| --- | --- | --- | --- |
|  | MVI (-), (*n*=12) | MVI (+), (*n*=12) | *p*-Intra |
| Age, years* | 61.50 (9.84) | 65.17 (9.43) | 0.361 |
| Gender |  |  | 0.027 |
| Female | 1 (8.3) | 7 (58.3) |  |
| Male | 11 (91.7) | 5 (41.7) |  |
| HBV |  |  | 0.400 |
| Negative | 6 (50.0) | 9 (75.0) |  |
| Positive | 6 (50.0) | 3 (25.0) |  |
| AFP |  |  | 1.000 |
| ＜20ng/ml | 12 (100.0) | 11 (8.3) |  |
| ≥20ng/ml | 0 (0.0) | 1 (91.7) |  |
| CEA |  |  | 0.590 |
| ＜5ng/ml | 11 (91.7) | 9 (75.0) |  |
| ≥5ng/ml | 1 (8.3) | 3 (25.0) |  |
| CA199 |  |  | 0.667 |
| ＜34U/ml | 7 (58.3) | 9 (75.0) |  |
| ≥34U/ml | 5 (41.7) | 3 (25.0) |  |
| Edmondson-Steiner grade |  |  | 0.684 |
| I-II | 7 (58.3) | 5 (41.7) |  |
| III-IV | 5 (41.7) | 7 (58.3) |  |
| Tumor size, mm* | 39.55 (14.53) | 61.79 (32.76) | 0.048 |
| Tumor morphology |  |  | 0.091 |
| (Hemi-)spherical and oval | 2 (16.7) | 3 (25.0) |  |
| Lobulated | 9 (75.0) | 4 (33.3) |  |
| Irregular | 1 (8.3) | 5 (41.7) |  |
| SI on T1WI |  |  | 1.000 |
| Low | 12 (100.0) | 12 (100.0) |  |
| Moderate | 0 (0.0) | 1 (0.0) |  |
| High | 0 (0.0) | 1 (0.0) |  |
| SI on T2WI-FS |  |  | 1.000 |
| Low | 0 (0.0) | 0 (0.0) |  |
| Moderate | 0 (0.0) | 0 (0.0) |  |
| High | 12 (100.0) | 12 (100.0) |  |
| Target sign on T2WI-FS |  |  | 0.214 |
| Negative | 3 (25.0) | 7 (58.3) |  |
| Positive | 9 (75.0) | 5 (41.7) |  |
| Target sign on DWI |  |  | 1.000 |
| Negative | 4 (33.3) | 4 (33.3) |  |
| Positive | 8 (66.7) | 8 (66.7) |  |
| Rim enhancement on AP |  |  | 1.000 |
| Negative | 1 (8.3) | 1 (8.3) |  |
| Positive | 11 (91.7) | 11 (91.7) |  |
| Complete rim on AP |  |  | 0.030 |
| Negative | 3 (27.3) | 9 (81.8) |  |
| Positive | 8 (72.7) | 2 (18.2) |  |
| Enhancement pattern |  |  | 0.488 |
| Gradual and filling | 10 (83.4) | 11 (91.7) |  |
| Arterial and persistent | 1 (8.3) | 0 (0.0) |  |
| Wash-in and wash-out | 1 (8.3) | 1 (8.3) |  |
| LI-RADS |  |  | 1.000 |
| LR-5 | 1 (8.3) | 1 (8.3) |  |
| LR-M | 11 (91.7) | 11 (91.7) |  |
| Intrahepatic duct dilatation |  |  | 0.241 |
| Negative | 7 (58.3) | 3 (25.0) |  |
| Positive | 5 (41.7) | 9 (75.0) |  |
| Hepatic capsular retraction |  |  | 1.000 |
| Negative | 8 (66.7) | 8 (66.7) |  |
| Positive | 4 (33.3) | 4 (33.3) |  |
| Visible vessel penetration |  |  | 0.317 |
| Negative | 11 (91.7) | 8 (66.7) |  |
| Positive | 1 (8.3) | 4 (33.3) |  |
| Peripherally hepatic enhancement |  |  | 0.684 |
| Negative | 7 (58.3) | 5 (41.7) |  |
| Positive | 5 (41.7) | 7 (58.3) |  |

**Supplementary Table 2.** Gd-DTPA MR imaging sequences and parameters.

| Parameter | T2WI-FS | DWI | IP-OP T1WI | 3D-VIBE T1WI |
| --- | --- | --- | --- | --- |
| Repetition time (msec) | 3500 | 3200 | 230 | 4.38 |
| Echo time (msec) | 84 | 56 | 2.38 and 4.76 | 1.93 |
| Section thickness (mm) | 5.5 | 5.5 | 5.5 | 3-4 |
| Matrix size | 320×224 | 128×128 | 320×240 | 320×240 |
| Field of view (mm^2^) | 380×308 | 380×308 | 380×278 | 380×297 |
| Gap (mm) | 1.1 | 1.1 | 1.1 | 0 |
| Average | 1 | 1 | 1 | 1 |

**Supplementary Table 3.** The feature numbers of each single MR sequence during the procedure of feature selection.

| Sequences | Input features | Intra-class correlation coefficient (≥0.75) | Variance threshold (0.8) | SelectKBest (*p*≤0.05) | LASSO selection |
| --- | --- | --- | --- | --- | --- |
| DWI | 2600 | 2579 | 2514 | 36 | 22 |
| T2 | 2600 | 2579 | 2566 | 31 | 12 |
| T1 | 2600 | 2578 | 2114 | 105 | 17 |
| T1A | 2600 | 2581 | 2568 | 46 | 9 |
| T1V | 2600 | 2566 | 2545 | 12 | 12 |
| T1D | 2600 | 2581 | 2560 | 6 | 5 |

**Supplementary Table 4.** The Rad-scores of each MR sequence in training and validation cohorts.

| Rad-score | Training cohort (*n*=130) | | | Validation cohort (*n*=33) | | | *p*-Inter |
| --- | --- | --- | --- | --- | --- | --- | --- |
|  | MVI (-), (*n*=92) | MVI (+), (*n*=38) | *p*-Intra | MVI (-), (*n*=23) | MVI (+), (*n*=10) | *p*-Intra |  |
| DWI | 0.20 (0.19) | 0.51 (0.19) | <0.001 | 0.25 (0.25) | 0.47 (0.23) | **0.025** | <0.001 |
| T2 | 0.23 (0.15) | 0.45 (0.22) | <0.001 | 0.34 (0.20) | 0.27 (0.16) | 0.361 | <0.001 |
| T1 | 0.24 (0.14) | 0.43 (0.16) | <0.001 | 0.26 (0.11) | 0.41 (0.14) | **0.003** | 0.054 |
| T1A | 0.26 (0.13) | 0.37 (0.12) | <0.001 | 0.31 (0.16) | 0.31 (0.14) | 0.944 | <0.001 |
| T1V | 0.24 (0.17) | 0.41 (0.18) | <0.001 | 0.31 (0.14) | 0.38 (0.14) | 0.156 | <0.001 |
| T1D | 0.25 (0.15) | 0.39 (0.15) | <0.001 | 0.29 (0.10) | 0.43 (0.09) | **0.001** | 0.371 |

**Supplementary Table 5.** The detailed information of radiomics features in diffusion-weighted image, pre-contrast T1-weighted image and delayed phase image.

| Sequences | Classes | Filters | Features | Coefficients |
| --- | --- | --- | --- | --- |
| DWI | first order | original | kurtosis | 0.000 |
| DWI | first order | normalize | kurtosis | -0.052 |
| DWI | glcm | log-sigma-0.5-mm | maximum probability | 0.103 |
| DWI | glcm | wavelet-lhl | correlation | 0.082 |
| DWI | glcm | wavelet-hlh | imc1 | 0.056 |
| DWI | glcm | speckle-noise | idmn | 0.018 |
| DWI | glcm | log-sigma-0.5-mm-3d | id | -0.156 |
| DWI | glcm | log-sigma-0.5-mm-3d | imc1 | -0.170 |
| DWI | gldm | wavelet-lhh | dependence variance | 0.119 |
| DWI | gldm | speckle-noise | small dependence low gray level emphasis | 0.005 |
| DWI | gldm | log-sigma-0.5-mm-3d | dependence variance | -0.003 |
| DWI | glrlm | wavelet-lhl | long run low gray level emphasis | 0.097 |
| DWI | glrlm | log-sigma-0.5-mm-3d | long run low gray level emphasis | -0.088 |
| DWI | glrlm | log-sigma-0.5-mm-3d | long run emphasis | -0.160 |
| DWI | glszm | log-sigma-0.5-mm-3d | gray level nonuniformity normalized | 0.143 |
| DWI | glszm | box-sigma-image | small area low gray level emphasis | 0.096 |
| DWI | glszm | wavelet-hhh | gray level nonuniformity normalized | 0.069 |
| DWI | glszm | log-sigma-0.5-mm-3d | size zone nonuniformity normalized | -0.020 |
| DWI | glszm | wavelet-lhh | size zone nonuniformity normalized | -0.042 |
| DWI | glszm | wavelet-lhl | size zone nonuniformity normalized | -0.044 |
| DWI | ngtdm | log-sigma-0.5-mm-3d | busyness | 0.046 |
| DWI | ngtdm | log-sigma-0.5-mm-3d | contrast | 0.011 |
| T1 | first order | log-sigma-0.5-mm-3d | skewness | 0.039 |
| T1 | glcm | wavelet-llh | imc1 | 0.080 |
| T1 | glcm | wavelet-lhl | joint energy | 0.013 |
| T1 | glcm | box-sigma-image | idmn | 0.004 |
| T1 | gldm | laplacian sharpening | large dependence low gray level emphasis | 0.035 |
| T1 | gldm | binomial blurimage | low gray level emphasis | 0.012 |
| T1 | gldm | binomial blurimage | large dependence low gray level emphasis | 0.010 |
| T1 | gldm | log-sigma-2-mm-3d- | dependence nonuniformity normalized | 0.003 |
| T1 | glrlm | mean | long run low gray level emphasis | 0.000 |
| T1 | glrlm | normalize | short run low gray level emphasis | -0.035 |
| T1 | glrlm | normalize | short run emphasis | -0.054 |
| T1 | glszm | log-sigma-0.5-mm-3d | small area low gray level emphasis | 0.032 |
| T1 | glszm | wavelet-hll | gray level nonuniformity normalized | 0.017 |
| T1 | glszm | log-sigma-0.5-mm-3d | low gray level zone emphasis | 0.014 |
| T1 | glszm | log-sigma-0.5-mm-3d | gray level nonuniformity normalized | 0.007 |
| T1 | glszm | wavelet-hhl | gray level nonuniformity normalized | 0.003 |
| T1 | glszm | wavelet-hhh | small area low gray level emphasis | -0.036 |
| T1D | first order | log-sigma-0.5-mm-3d | median | 0.067 |
| T1D | glcm | wavelet-hhl | imc1 | 0.119 |
| T1D | glcm | wavelet-hhl | imc2 | 0.044 |
| T1D | glcm | wavelet-hhh | correlation | -0.050 |
| T1D | glszm | wavelet-hlh | gray level nonuniformity normalized | 0.083 |

Details and formulas of features:

<https://pyradiomics.readthedocs.io/en/latest/features.html#module-radiomics.firstorder>


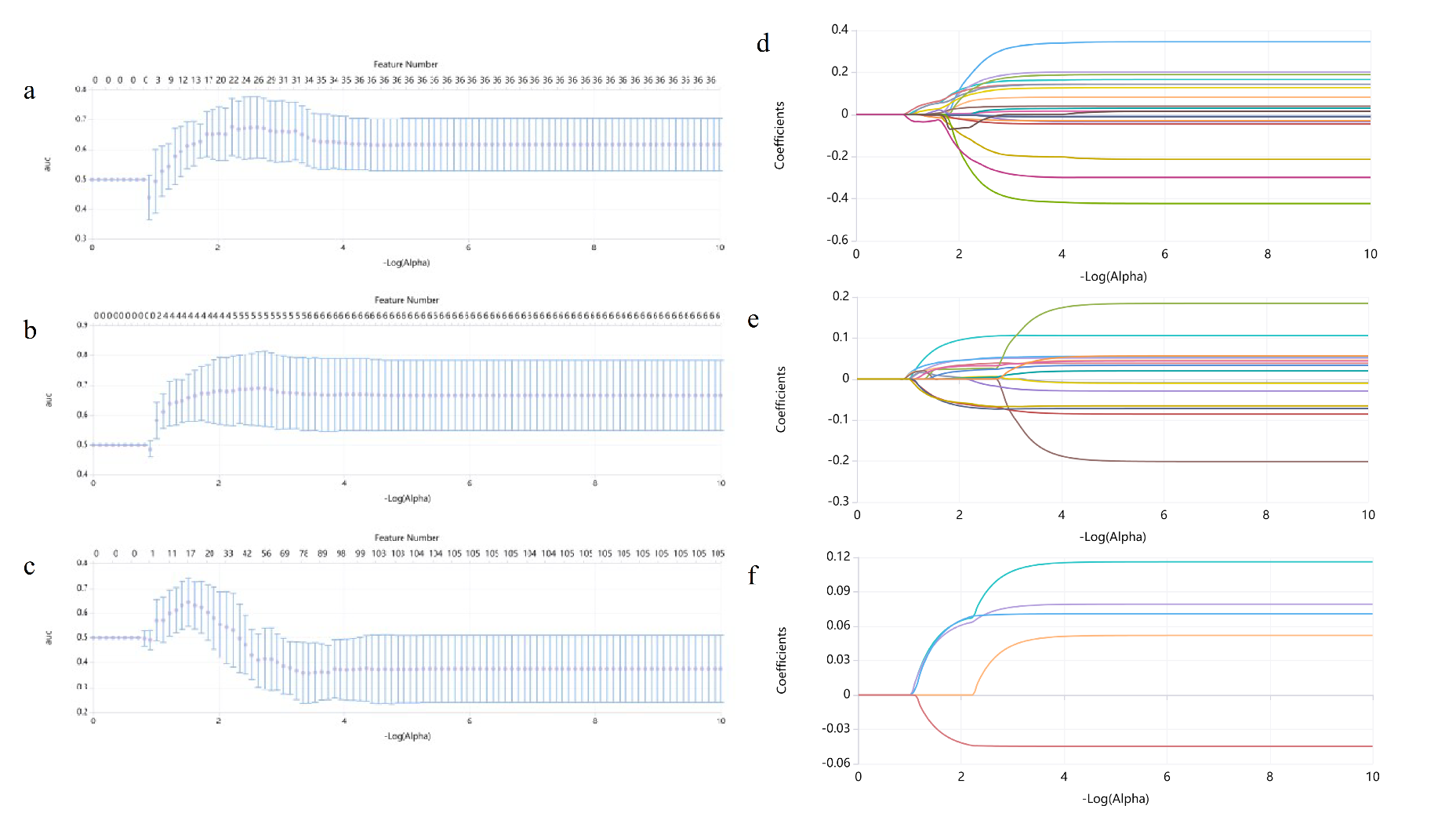


**Supplementary Figure 1.** Radiomics feature selection using the least absolute shrinkage and selection operator (LASSO) regression model on diffusion-weighted image (a, d), pre-contrast T1-weighted image (b, e), and delayed phase image (c, f).


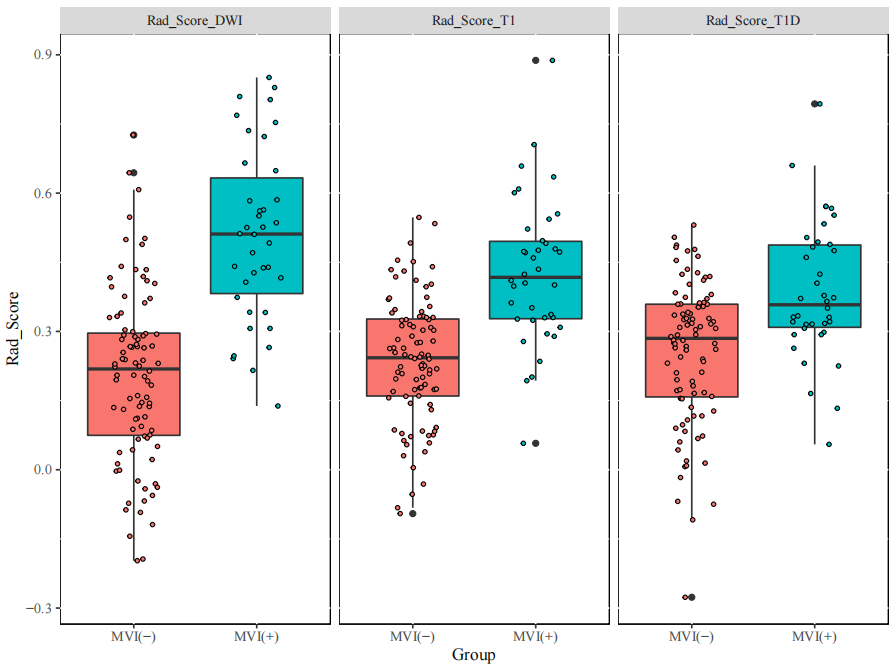


**Supplementary Figure 2.** The Rad-scores of diffusion-weighted image, pre-contrast T1-weighted image, and delayed phase image between MVI-negative and MVI-positive groups.


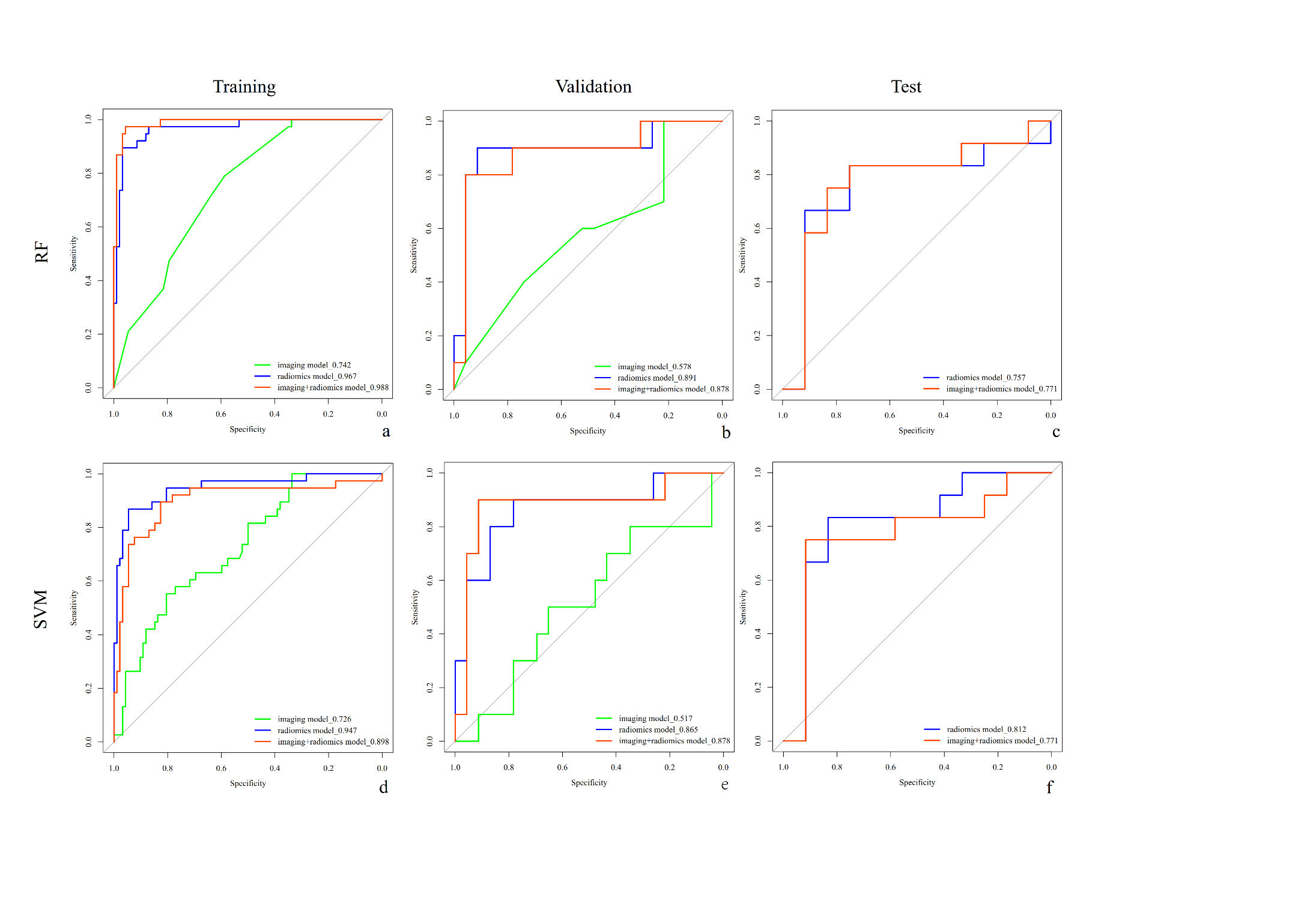


**Supplementary Figure 3.** Comparison of receiver operating characteristics (ROC) curves for prediction of MVI in ICC by random forest (RF) and support vector machine (SVM). ROC curves of imaging model, radiomics model and MVI prediction model in the training (a, d), validation (b, e) and test (c, f) cohort.
